# MEASURING SOCIAL ISOLATION AMONG US-BORN AND FOREIGN-BORN OLDER ADULTS

**DOI:** 10.1093/geroni/igad104.0496

**Published:** 2023-12-21

**Authors:** Rita Hu, Noah Webster, Kristine Ajrouch, Toni Antonucci

**Affiliations:** University of Michigan, Ann Arbor, Michigan, United States; University of Michigan, Ann Arbor, Michigan, United States; Eastern Michigan University, Ypsilanti, Michigan, United States; University of Michigan, Ann Arbor, Michigan, United States

## Abstract

While older immigrants are at increased risk of social isolation, little research has examined if established measures are equivalent across nativity groups. This study evaluates the measurement invariance of objective and subjective social isolation between US-born and foreign-born older adults. Data are from the Detroit Area Wellness Network (DAWN) study and were collected via a telephone survey from three groups prominent in metro-Detroit: African Americans, Middle Eastern/Arab Americans, and Non-Hispanic White Americans (N=611; Mage = 72.7 [64-98]). The sample was comprised of 29% immigrants. Objective social isolation was measured by network size, household size, and contact frequency with network members. Subjective social isolation was measured by feeling lonely, relationship quality, frequency of arguing, and frequency of conflict. Confirmatory Factor Analysis (CFA) was used to evaluate the dimensional structure, and multiple-group CFA was used to test for measurement variance by immigrant status. The measurement invariance models of objective social isolation showed a good fit after allowing the intercepts of both network and household size to vary in the scalar model. The measurement invariance models of subjective social isolation showed a good fit after allowing the estimate and intercept of loneliness and the intercept of argument to vary in the scalar model. Establishing measurement invariance in both objective and subjective social isolation is essential for valid interpretation of group differences among US- and foreign-born adults in aspects of social isolation. Findings show indicators of social network structure and perceived relationship quality may be useful to examine social isolation among older immigrants.

